# Effect of epidural block on surgical conditions during pediatric subumbilical laparoscopic surgery involving a supraglottic airway: a randomized clinical trial

**DOI:** 10.3389/fmed.2023.1250039

**Published:** 2023-10-06

**Authors:** Lei Wu, Siwei Wei, Zhen Xiang, Eryou Yu, Zheng Chen, Zhen Du, Shuang Quan Qu

**Affiliations:** Department of Anesthesiology, Hunan Children’s Hospital, Changsha, China

**Keywords:** supraglottic airway, laryngeal mask airway, neuromuscular block, epidural block, pediatric laparoscopy, surgical conditions

## Abstract

**Background:**

Few studies have examined the effect of epidural block on surgical conditions during pediatric subumbilical laparoscopic surgery involving a supraglottic airway (SGA). This study investigated the surgical condition scores for such procedures in cases where neuromuscular block, epidural block, or neither was used.

**Methods:**

A total of 150 patients aged 3–12 years undergoing laparoscopic orchiopexy with a ProSeal SGA device were randomly allocated to one of three groups: the control group (did not receive neuromuscular block and epidural block), the NMB group [received a neuromuscular block (train-of-four 1–2 twitches) using rocuronium], or the EDB group (received an epidural block using ropivacaine). The primary outcome was the quality of surgical conditions evaluated with the Leiden-Surgical Rating Scale by the blinded surgeon. The secondary outcome measures included intraoperative hemodynamic data (including mean arterial pressure and heart rate), the SGA device removal time, the PACU discharge time, the pain score in the PACU and intraoperative adverse events (including bradycardia, hypotension, peak airway pressure > 20 cmH_2_O, and poor or extremely poor surgical conditions occurred during the operation). Statistical analysis was performed with one-way analysis of variance, the Kruskal–Wallis test, the chi-square test or Fisher’s exact test. Bonferroni corrections for multiple comparisons were made for primary and secondary outcomes.

**Results:**

Surgical condition scores were significantly higher in the NMB and EDB groups than in the control group (median difference: 0.8; 95% confidence interval [CI], 0.5–1.0; *p* < 0.0001; and median difference: 0.7; 95% CI, 0.5–0.8; *p* < 0.0001, respectively). Blood pressure and heart rate were significantly lower in the EDB group than in the other two groups (*p* < 0.0001 and *p* = 0.004). Patients in the EDB group had significantly lower pain scores during PACU than those in the other two groups (*p* < 0.0001). The sufentanil dose was lower in the EDB group than in the other two groups (*p* = 0.001).

**Conclusion:**

Epidural block can improve surgical conditions during pediatric subumbilical laparoscopic surgery involving a SGA to a degree comparable to that with moderate neuromuscular block.

## Introduction

Supraglottic airways (SGAs) have been shown to be a safe alternative to endotracheal intubation in pediatric subumbilical laparoscopic surgical procedures (1–4). Improvement of surgical conditions is also particularly important in pediatric laparoscopic surgery involving a SGA (4). Our previous study demonstrated the important role of neuromuscular block in the optimization of conditions for this type of surgery (4). However, neuromuscular block agents are a double-edged sword because these agents may improve anesthesia and surgical conditions while increasing the risk of postoperative complications and procedural delay, especially in ambulatory or day-case surgery (5, 6). Therefore, increasing the depth of neuromuscular block in these procedures to obtain better surgical conditions is concerning. Epidural block, which can reduce the amount of general anesthesia drugs and relieve postoperative pain, is widely used in pediatric subumbilical surgery (7–10). Furthermore, epidural block can not only eliminate sensation but also relax the muscles in the blockade area (11). At present, few investigations have been conducted to study the effect of epidural block on surgical conditions during pediatric laparoscopic surgery. The aims of this study were to determine whether an epidural block was capable of improving surgical conditions when a SGA device was used in pediatric subumbilical laparoscopic surgery without the use of neuromuscular blocks.

## Methods

### Study design

This study was a prospective, single-center, parallel, double-blind, randomized clinical trial. This study was approved by the institutional review board at the Hunan Children’s Hospital (HCHLL-2021-75, approved on September 27, 2021) and written informed consent was obtained from all subjects participating in the trial. The trial was registered prior to patient enrollment at Chinese Clinical Trial Registry (ChiCTR2200061405, Principal investigator: L. W., Date of registration: June 23, 2022). The trial was performed between June 2022 and February 2023 at Hunan Children’s Hospital (Hunan, China).

### Inclusion and exclusion criteria

We recruited patients aged 3–12 years with American Society of Anesthesiologists (ASA) physical status class I or II scheduled for elective laparoscopic orchiopexy surgery. Patients with previous intra-abdominal surgery, ASA physical status class ≥3, known or suspected neuromuscular disease, bronchial asthma, allergy to medication to be used during anesthesia, severe obesity, contraindications for epidural puncture, congenital heart disease, congenital airway malformation, or developmental delay were excluded from the study. Potential participants were first identified from the list of elective surgeries by a research team member. The research team then contacted the parents or guardians of the potential participants before surgery and verbally explained the details of the study and the possible consequences. Written informed consent was obtained from the parents or guardians of all participants. All parents or guardians provided written informed consent for their children’s participation in the study and informed assent from the patient when applicable.

### Randomization and masking

All subjects were blinded to the treatment. All surgical procedures and scoring of surgical conditions in this study were performed by the same surgeon who was blinded to the treatment. Similarly, the research assistants who recorded or statistically analyzed the data were blinded to the treatment. Participants were randomly assigned to the control group, epidural block group, or neuromuscular block group using a computer-generated random number table. The codes of the participants were concealed in sealed, opaque envelopes that were only presented to the anesthesia team on the day of surgery. Interventions for all subjects were performed by an anesthesiologist who was not involved in the study. The surgeons were not allowed in the operating room until anesthesia induction and intervention had been completed to ensure that they were blinded to the treatment. Moreover, during surgery, the drape was used to blind the surgeons to the TOF-watch monitor.

### General anesthesia protocol

Standard monitoring (pulse oximetry, capnography, electrocardiography, and noninvasive blood pressure) was used for patients in all the groups after entering the operating room. Anesthesia induction was performed by intravenous injection of propofol 2–5 mg/kg and sufentanil 0.2 μg/kg. After the drugs were administered, a ProSeal (Hisern, Zhejiang, China) SGA device was used to secure the airway. During the operation, tidal volume was set to 10 mL/kg, gas flow was set to 2 L/min, and ventilatory frequency was set according to the subject’s age or EtCO_2_. Propofol was used for anesthesia maintenance to maintain the bispectral index (BIS) at 40–60. Sufentanil was added intraoperatively based on changes in blood pressure and heart rate. A single bolus of sufentanil 0.1–0.2 μg/kg was administered at the discretion of the attending anesthesiologist if a 10% increase in blood pressure and/or heart rate occurred during the operation.

### Groups

Patients were randomly assigned to the control, neuromuscular block or epidural block group. Subjects in the control group did not receive epidural block or any neuromuscular block agents administered intravenously during anesthesia and surgery. In the neuromuscular block group (NMB group), an intravenous bolus dose of 0.6 mg/kg rocuronium was given for intubation, followed by a continuous infusion of rocuronium to maintain the train-of-four (TOF) count at 1 to 2 twitches. The TOF watch (TOF-watch SX, MSD BV, Oss, the Netherlands) was calibrated before neuromuscular block monitoring was performed. In the epidural block group (EDB group), patients received a single epidural block. The epidural puncture site was placed at the level of the thoracolumbar transition (Th12-L1) (10). After anesthesia induction, the children were turned to a left lateral position with the hips and knees flexed. Palpation was used to determine the puncture site. Sterile preparation was followed by an initial ultrasound scan of the puncture area and the dura mater. Then, epidural puncture was performed under ultrasound guidance using the loss of resistance with the saline technique before injecting ropivacaine into the epidural space. In accordance with our clinical standards and previous studies (8, 10, 12), 0.5 mL/kg ropivacaine 3.8 mg/mL was injected into the epidural space.

The surgeons were not allowed to enter the operating room until the attending anesthesiologist had completed all anesthetic procedures. During the operation, the CO_2_ insufflation pressure was maintained at 8 cm H_2_O. When poor or extremely poor surgical conditions occurred, a bolus dose of rocuronium 0.3 mg/kg was administered immediately, irrespective of the group. In the NMB group, a single bolus of sugammadex 2 mg/kg i.v. was administered at the end of surgery, and the SGA device was removed when the TOF ratio was >0.9. In the control and EDB groups, the SGA device was removed after the subjects returned to spontaneous respiration.

### Outcome measures

The primary outcome of this study was the surgical condition score assessed by the surgeon according to the Leiden-Surgical Rating Scale (L-SRS) during pneumoperitoneum (4, 13, 14). The L-SRS quantifies surgical conditions based on visibility, surgical space, muscle contraction, handling tactics, and patient movement. The L-SRS ranks surgical conditions on a 5-point scale from extremely poor conditions to optimal conditions: extremely poor (score = 1), poor (score = 2), acceptable (score = 3), good (score = 4), and excellent (score = 5) surgical working conditions (Table 1). The surgeon evaluated and scored the surgical conditions every 10 min after CO_2_ insufflation. Each subject’s final surgical condition score was the mean of all 10 min L-SRS values. The secondary outcome measures included intraoperative hemodynamic data, the SGA device removal time, the PACU discharge time, the pain score during PACU and intraoperative adverse events. Among them, intraoperative hemodynamic data included mean arterial pressure (MAP) and heart rate (HR), and intraoperative adverse events included bradycardia, hypotension, peak airway pressure (Ppeak) > 20 cmH_2_O, and poor or extremely poor surgical conditions occurred during the operation. Table 1 shows the definitions of outcome measures.

**Table 1:** Definition of outcome measures.

	Definition
Leiden-surgical rating scale (L-SRS)	
Extremely poor (score = 1)	The surgeon is unable to work because of coughing or because of the inability to obtain a visible laparoscopic field because of inadequate muscle relaxation. Additional neuromuscular blocking agents must be given
Poor (score = 2)	There is a visible laparoscopic field, but the surgeon is severely hampered by inadequate muscle relaxation with continuous muscle contractions, movements, or both with the hazard of tissue damage. Additional neuromuscular blocking agents must be given
Acceptable (score = 3)	There is a wide visible laparoscopic field but muscle contractions, movements, or both occur regularly causing some interference with the surgeon’s work. There is the need for additional neuromuscular blocking agents to prevent deterioration
Good (score = 4)	There is a wide laparoscopic working field with sporadic muscle contractions, movements, or both. There is no immediate need for additional neuromuscular blocking agents unless there is the fear of deterioration
Excellent (score = 5)	There is a wide visible laparoscopic working field without any movement or contractions. There is no need for additional neuromuscular blocking agents
Secondary outcomes	
SGA device removal time	Time from cessation of propofol administration to removal of the SGA device
PACU discharge time	Time from PACU admission until a modified Aldrete scale score ≥ 9 was achieved
Hypotension	More than 30% decrease in mean arterial pressure from baseline for >5 min
Bradycardia	More than 20% decrease in the baseline heart rate for >30 s

### Sample size and statistical analysis

The minimum clinically relevant mean difference between treatment groups on the 5-point L-SRS scale was 0.5 points. The results in our pilot study showed that the mean (SD) L-SRS scores were 3.4 (0.6), 4.1 (0.5), and 4.0 (0.5) in the control group, NMB group and EDB group, respectively. Thirty-nine subjects per group was predetermined based on sample size calculation to achieve 90% power at α = 0.05 to detect a 0.5-point difference in mean L-SRS for an expected SD of 0.6. To allow for subject withdrawal or loss of patients for other reasons, the sample size was enlarged to 50 per group. Data distribution was assessed for normality using the Kolmogorov–Smirnov. Continuous variables are expressed as the mean [standard deviation (SD)] or median [interquartile range (IQR)]. Categorical variables are expressed as the number (percentage). Comparisons of normally distributed continuous variables were performed using one-way analysis of variance (ANOVA). Comparisons of nonnormally distributed continuous variables, such as L-SRS scores, were performed using the nonparametric Kruskal–Wallis test. Comparisons of categorical data were performed using the chi-square test. A *p* value <0.05 was considered statistically significant. *Post hoc* pairwise comparisons were performed with Bonferroni corrections. For multiple comparisons of the outcomes, the significance threshold was set to be *p* < 0.017 (0.05/3) because 3 comparisons were performed (EDB versus Control, EDB versus NMB, and NMB versus Control). Statistical analyses were performed using IBM SPSS Statistics 26.0 (SPSS Inc., Chicago, IL, USA).

## Results

**Figure 1:**
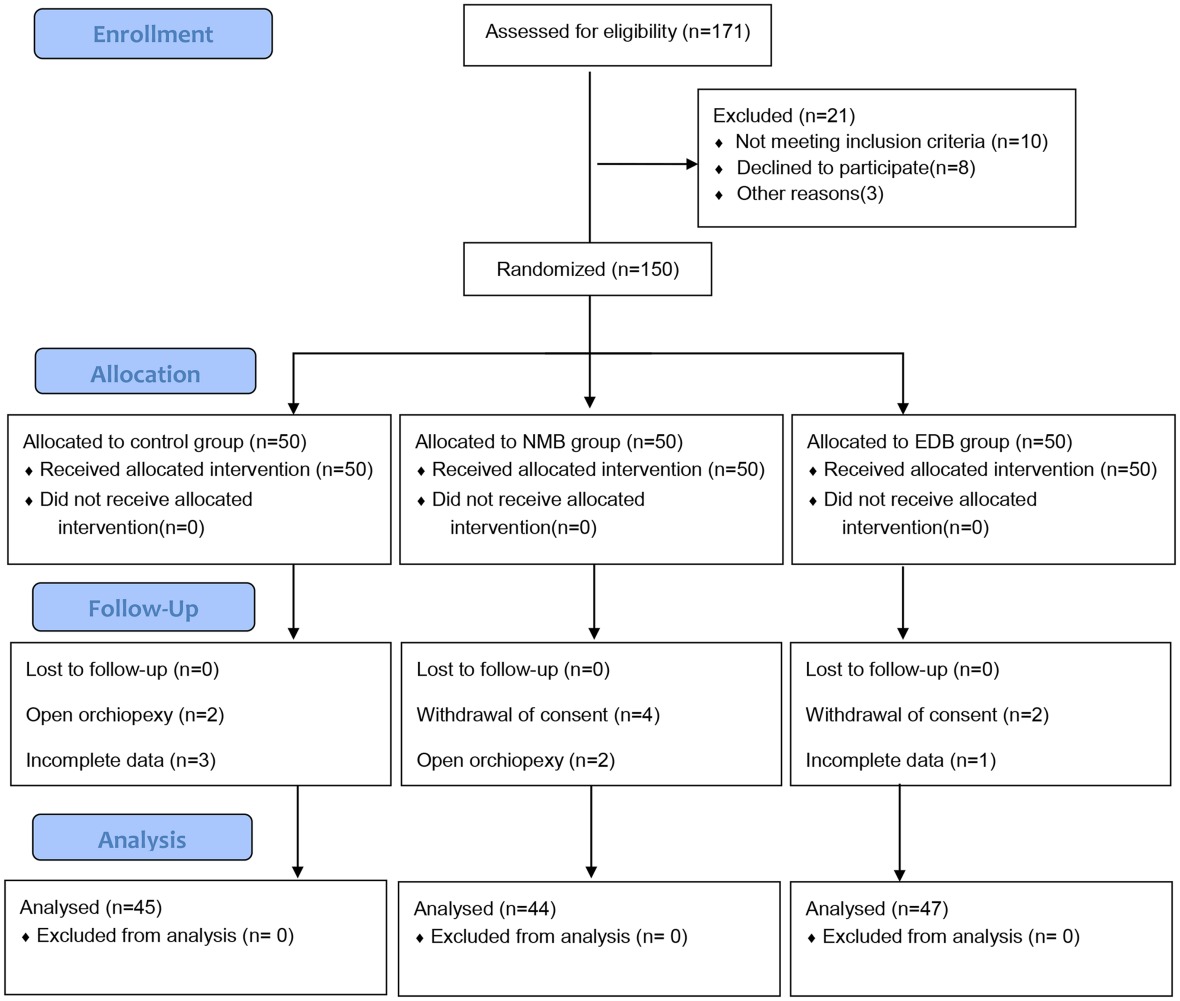
Consort flow diagram.

A total of 150 children participated in this randomized study from June 2022 to February 2023. Fourteen of the children were excluded due to withdrawal of consent, a change in procedure or loss of data. Ultimately, 136 participants completed the study and were included in the analysis. Figure 1 shows the patient recruitment profile and study flow. There were no significant differences in baseline characteristics between the three groups (Table 2).

**Table 2:** Characteristics of the patient groups.

	Control (*n* = 45)	NMB (*n* = 44)	EDB (*n* = 47)
Median age, yr (range)	5.2 (3.1–11.1)	5.1 (3.2–10.6)	5.1 (3.1–11.3)
Weight (kg)	20.3 ± 5.9	19.9 ± 5.5	20.6 ± 6.6
Height (cm)	113.6 ± 14.4	112.4 ± 13.2	115.1 ± 15.0
BMI (kg m^−2^)	15.4 ± 1.1	15.4 ± 1.2	15.1 ± 1.1
Baseline MAP (mmHg)	70.0 ± 3.5	71.2 ± 3.2	70.8 ± 3.4
Baseline HR (min^−1^)	92.6 ± 8.2	93.5 ± 8.3	92.7 ± 8.5

Data are presented as mean ± SD or median (range). NMB, neuromuscular block; EDB, epidural block; BMI, body mass index; MAP, mean arterial pressure; HR, heart rate.

**Figure 2:**
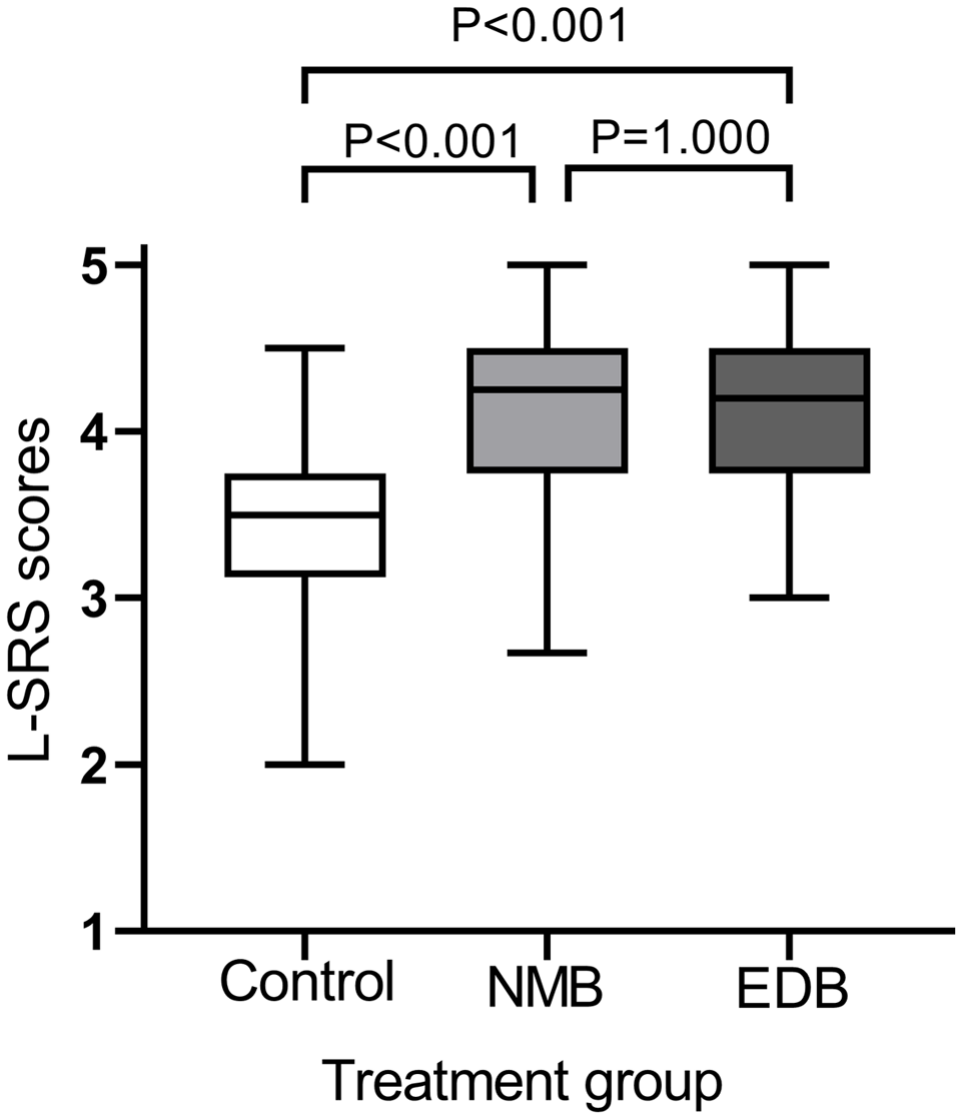
Surgical ratings by the surgeon during pneumoperitoneum using the 5-point Leiden-surgical rating scale (L-SRS). Violin plots showing differences in surgical condition scores between groups (*p* < 0.0001, Kruskal-Wallis test). The results of pairwise comparisons were as follows (Mann–Whitney *U* test and Bonferroni-adjusted *p* values): control group vs. NMB group, *p* < 0.001; control group vs. EDB group, *p* < 0.001; NMB group vs. EDB group, *p* = 1.000.

Figure 2 shows that surgical condition scores were different in the study groups (*p* < 0.0001). The median (IQR) surgical condition scores were 3.5 (3.1–3.8) in the control group, 4.3 (3.8–4.5) in the NMB group, and 4.2 (3.8–4.5) in the EDB group. The pairwise comparison results showed that the control group vs. NMB group (median difference: 0.8, 95% CI, 0.5–1.0; *p* < 0.0001) and control group vs. EDB group (median difference: 0.7, 95% CI, 0.5–0.8, *p* < 0.0001) had significant differences, while the NMB group vs. EDB group (*p* = 1.000) had no significant differences.

Table 3 shows the secondary outcome data and the analysis results. There were significant differences in intraoperative blood pressure (*p* < 0.0001) and heart rate (*p* = 0.004) among the three groups (Table 3). The intraoperative mean arterial pressure (MAP) in the EDB group was significantly lower than that in the other two groups (mean difference: −5.4; 95% CI, −7.3 – −3.5; *p* < 0.0001; and mean difference: −4.4; 95% CI, −6.6 – −2.2; *p* < 0.0001, respectively). Similarly, the intraoperative heart rate of patients in the EDB group was significantly lower than that in the other two groups (mean difference: −6.6; 95% CI, −10.9 – −2.3; *p* = 0.003; and mean difference: −5.2; 95% CI, −9.3 – −1.1; *p* = 0.014, respectively). Intraoperative rates of hypotension, bradycardia, Ppeak>20 cmH_2_O, and poor or extremely poor surgical conditions, as well as the mean time of SGA device removal and PACU discharge, were similar among the three groups (Table 3). Notably, patients in the EDB group had significantly lower pain scores during PACU than those in the other two groups (median difference: −2; 95% CI, −2 – −1; *p* < 0.0001; and median difference: −2; 95% CI, −2 – −1; *p* < 0.0001, respectively).

**Table 3:** Secondary endpoints of the three study groups.

	Control (*n* = 45)	NMB (*n* = 44)	EDB (*n* = 47)	*p*
Intraoperative MAP (mmHg)^d^	61.5 ± 3.4	60.4 ± 5.0	56.1 ± 5.5	<0.0001^a^
Intraoperative HR (min^−1^)^e^	93 ± 10	91 ± 9	86 ± 11	0.004^a^
Intraoperative hypotension, *n* (%)	3 (6.7)	2 (4.5)	7 (14.9)	0.237^b^
Intraoperative bradycardia, *n* (%)	0 (0)	0 (0)	1 (2.1)	1.000^b^
Ppeak>20 cmH_2_O, *n* (%)	6 (13.3)	3 (6.8)	5 (10.6)	0.644^b^
Poor or extremely poor surgical conditions, *n* (%)	3 (6.7)	1 (2.3)	0 (0)	0.169^b^
SGA device removal time (min)	4.6 ± 1.5	5.1 ± 1.5	4.7 ± 1.3	0.276^a^
PACU discharge time (min)	14.7 ± 2.5	14.9 ± 2.8	13.8 ± 2.4	0.077^a^
Pain score during PACU, median (IQR)^f^	3 (2.5–4)	3 (2.3–3.8)	1 (1–2)	<0.0001^c^

Data are expressed as mean ± SD, number (%) or median (IQR). *p* < 0.017 is set to be significant for the multiple comparisons. ANOVA, analysis of variance; NMB, neuromuscular block; EDB, epidural block; MAP, mean arterial pressure; HR, heart rate; Ppeak, peak airway pressure; SGA, supraglottic airway; PACU, post-anesthesia care unit.

^a^One-way ANOVA.

^b^Chi-square test.

^c^Kruskal-Wallis test.

^d^EDB group different from control group, *p* < 0.0001, and NMB group, *p* < 0.0001.

^e^EDB group different from control group, *p* = 0.003, and NMB group, *p* = 0.014.

^f^EDB group different from control group, *p* < 0.0001, and NMB group, *p* < 0.0001.

Table 4 shows the intraoperative measurement data and the analysis results. During surgery, the values of BIS, the dosages of propofol, and the time of pneumoperitoneum and operation were similar between treatments. The dosages of sufentanil were different in the study groups, and the sufentanil dose was lower in the EDB group than in the other two groups (mean difference: −1; 95% CI, −1.6 – −0.3; *p* = 0.0043; and mean difference: −1.4; 95% CI, −2.2 – −0.6; *p* = 0.0005, respectively). In the NMB group, the median (IQR) TOF count was 1 (1–2), and the mean (SD) doses of rocuronium and sugammadex were 19.8 (6.1) mg and 39.8 (10.9) mg, respectively.

**Table 4:** Measurements during surgery.

	Control (*n* = 45)	NMB (*n* = 44)	EDB (*n* = 47)	*p*
BIS	49 ± 5	48 ± 4	50 ± 4	0.088^a^
TOF count, median (rang)	–	1 (1–2)	–	–
Sufentanil (μg)^b^	5.3 ± 1.7	5.8 ± 2.3	4.4 ± 1.4	0.001^a^
Propofol (mg)	124.5 ± 39.4	118.9 ± 35.7	125.8 ± 44.2	0.689^a^
Rocuronium (mg)	–	19.8 ± 6.1	–	–
Sugammadex (mg)	–	39.8 ± 10.9	–	–
Pneumoperitoneum time (min)	37.9 ± 6.2	35.5 ± 6.1	37.8 ± 6.7	0.123^a^
Operation time (min)	52.2 ± 7.4	49.3 ± 7.2	51.7 ± 7.6	0.162^a^

Data are expressed as mean ± SD or median (range). ANOVA, analysis of variance; NMB, neuromuscular block; EDB, epidural block; BIS, bispectral index; TOF, train-of-four.

^a^One-way ANOVA.

^b^EDB group different from control group, *p* = 0.0043, and NMB group, *p* = 0.0005.

## Discussion

This study demonstrated that epidural block significantly improved the surgical conditions for pediatric subumbilical laparoscopic surgery involving a SGA. Compared with moderate neuromuscular block, epidural block showed a similar optimization effect on surgical conditions in this type of surgery. Although the EDB group had a lower intraoperative blood pressure and heart rate than the other two groups, the incidence of intraoperative hypotension and bradycardia was similar in the three groups. Importantly, epidural block was able to significantly reduce the intraoperative opioid dosage and provide patients with better postoperative analgesia.

In previous anesthesia procedures, neuromuscular blockers seemed to be the only option to optimize surgical conditions for laparoscopic surgery (4, 13–21). Blobner and colleagues (18) reported that deep neuromuscular block ameliorated surgical conditions for laparoscopic cholecystectomy compared with no neuromuscular block. Madsen and colleagues (21) also found that deep neuromuscular block enlarged surgical space in gynaecologic laparoscopy surgery compared with no neuromuscular block. In children, our previous research showed that moderate neuromuscular block improved surgical conditions for laparoscopic hernia repair compared with no neuromuscular block (4). Currently, with the development of anesthesia techniques, the SGA is increasingly used in laparoscopic surgery in children (1–4, 22, 23). Nevertheless, the use of neuromuscular blockers to improve surgical conditions in this type of surgery, especially in ambulatory or day-case surgery, is controversial (22, 23). Our previous research has demonstrated the important role of neuromuscular block in the optimization of conditions for short-duration pediatric laparoscopic surgery involving a SGA (4). Worryingly, neuromuscular block may cause postoperative complications and procedural delay (5, 6, 24–26). Moreover, use of neuromuscular block agents during anesthesia has been repeatedly shown to increase the risk of both immediate and longer term postoperative pulmonary complications. In a prospective observational study, Kirmeier and colleagues (27) reported that perioperative use of neuromuscular block agents was associated with an increased risk of postoperative pulmonary complications and that use of a reversal agent did not decrease that risk. These findings were further supported by the results in the study by Hammer and colleagues (28). Hence, the use of neuromuscular blockers in pediatric ambulatory or day-case laparoscopic surgery involving a SGA requires weighing the advantages and disadvantages.

Therefore, in pediatric laparoscopic surgery, we attempted to optimize surgical conditions by using epidural block instead of neuromuscular block. In this study, we evaluated the effect of an epidural block on the conditions during pediatric subumbilical laparoscopic surgery involving the SGA and found that the epidural block showed a similar optimization effect on surgical conditions compared with that of a moderate neuromuscular block. The maintenance of abdominal pressure mainly depends on the tension of the abdominal wall muscles. Zarick and colleagues (11) found that the abdominal muscles were blocked by a single injection of ropivacaine at the L2-3 level. This indicates that epidural block is feasible in optimizing the conditions of laparoscopic surgery in children. Thus, epidural block can replace neuromuscular block in ambulatory or day-case surgery to improve the surgical conditions for pediatric laparoscopic surgery involving the SGA, which can minimize postoperative complications and procedural delay caused by neuromuscular block. In fact, many anesthesiologists in our hospital use neuromuscular blockers in addition to epidural blocks during pediatric laparoscopic surgery involving a SGA. Their main concern was that the epidural block would not be sufficient to completely block the abdominal muscles, resulting in poor or extremely poor surgical conditions. It is clear from this study that neuromuscular blockers are not necessary in this type of surgery with an epidural block. Our previous study showed that a mean surgical condition score of 4.0 could be achieved in laparoscopic surgery in children under 3 years of age despite the absence of neuromuscular block (4). This may be due to their relatively weak muscle strength (29). Hence, in our study, we opted for children over 3 years of age to avoid false-negative results. Significantly, ropivacaine at a concentration of 3.8 mg/mL and a volume of 0.5 mL/kg was used in this study, which was based on our clinical standards as well as previous studies (8, 10, 12). Based on these, epidural block can indeed be used as a new method to optimize the conditions of laparoscopic surgery in children. Moreover, in this study, patients with epidural block had more stable haemodynamics during surgery. This was the reason that the dosage of sufentanil was significantly lower in the epidural block group than in the other two groups. Although the intraoperative MAP and heart rate were significantly lower in the EDB group than in the other two groups, the incidence of intraoperative hypotension and bradycardia was not significantly increased. In this study, seven patients (14.9%) with hypotension and one patient with bradycardia (2.1%) in the EDB group returned to normal levels with accelerated fluid rehydration or a single atropine injection without more serious cardiovascular adverse events. The intraoperative incidence of high peak airway pressure (Ppeak>20 cmH2O) was lower in the NMB group than in the other two groups (6.8% vs. 13.3% vs. 10.6%) but was not statistically significant. None of these patients had a peak airway pressure higher than 25 cm H_2_O, and no gas leakage was detected in them. The SGA device removal time and PACU discharge time were similar in all three groups, which is consistent with our previous study (4). The reason for this may be the use of a neuromuscular block reversal agent (sugammadex) in the patients of the NMB group. During PACU, pain scores in the EDB group were significantly lower than those in the other two groups. This is also one of the most important advantages of an epidural block in pediatric lower abdominal surgery (30).

The study has some limitations. First, it focused only on orchiopexy. Therefore, only male patients were included. Second, the epidural puncture point in this study was thoracolumbar, so the effect of a caudal block on the conditions of laparoscopic surgery in children cannot be elucidated. Therefore, further studies are needed to clarify the effects of caudal block on the surgical conditions of laparoscopic surgery in children. Third, attending anesthesiologists were not blinded to the grouping of the study, which may have led to bias for some secondary outcomes.

In summary, this study demonstrated the ability of epidural block to significantly improve surgical conditions during subumbilical laparoscopic surgery involving the SGA airway in children. This technique avoids the use of neuromuscular blocking agents in this type of surgery and minimizes postoperative complications caused by residual neuromuscular block.

## Data availability statement

The original contributions presented in the study are included in the article/supplementary material, further inquiries can be directed to the corresponding authors.

## Ethics statement

The studies involving humans were approved by Institutional Review Board at the Hunan Children’s Hospital. The studies were conducted in accordance with the local legislation and institutional requirements. Written informed consent for participation in this study was provided by the participants’ legal guardians/next of kin.

## Author contributions

All authors listed have made a substantial, direct, and intellectual contribution to the work and approved it for publication.

## References

[1] Kumar A Sinha C Kumar N Kumar A Kumar B Kumar A. Comparison of the oropharyngeal leak pressure between three second generation supraglottic airway devices during laparoscopic surgery in pediatric patients. Paediatr Anaesth. (2022) 32:843–850. doi: 10.1111/pan.14447 35338764

[2] Su MP Hu PY Lin JY Yang ST Cheng KI Lin CH. Comparison of laryngeal mask airway and endotracheal tube in preterm neonates receiving general anesthesia for inguinal hernia surgery: a retrospective study. BMC Anesthesiol. (2021) 21:195. doi: 10.1186/s12871-021-01418-2 34289809 PMC8293587

[3] Sinha A Sharma B Sood J. ProSeal as an alternative to endotracheal intubation in pediatric laparoscopy. Paediatr Anaesth. (2010) 17:327–32. doi: 10.1111/j.1460-9592.2006.02127.x 17359400

[4] Wu L Wei SW Xiang Z Yu EY Qu SQ Du Z. Effect of neuromuscular block on surgical conditions during short-duration paediatric laparoscopic surgery involving a supraglottic airway. Br J Anaesth. (2021) 127:281–8. doi: 10.1016/j.bja.2021.04.031, PMID: 34147245

[5] Schreiber JU. Management of neuromuscular blockade in ambulatory patients. Curr Opin Anaesthesiol. (2014) 27:583–8. doi: 10.1097/ACO.0000000000000134 25251920

[6] Cutter TW. What is the role of neuromuscular blocking drugs in ambulatory anesthesia? Curr Opin Anaesthesiol. (2002) 15:635–9. doi: 10.1097/00001503-200212000-00006, PMID: 17019264

[7] Öksüz G Arslan M Urfalıoğlu A Güler AG Tekşen Ş Bilal B. Comparison of quadratus lumborum block and caudal block for postoperative analgesia in pediatric patients undergoing inguinal hernia repair and orchiopexy surgeries: a randomized controlled trial. Reg Anesth Pain Med. (2020) 45:187–91. doi: 10.1136/rapm-2019-101027, PMID: 31907294

[8] Opfermann P Zadrazil M Tonnhofer U Metzelder M Marhofer P Schmid W. Ultrasound-guided epidural anesthesia and sedation for open transvesical Cohen ureteric reimplantation surgery in 20 consecutive children: a prospective case series and proof-of-concept study. Minerva Anestesiol. (2022) 88:564–72. doi: 10.23736/S0375-9393.22.15904-3, PMID: 35381834

[9] Veneziano G Tobias JD. Chloroprocaine for epidural anesthesia in infants and children. Paediatr Anaesth. (2017) 27:581–90. doi: 10.1111/pan.13134 28321983

[10] Opfermann P Marhofer P Springer A Metzelder M Zadrazil M Schmid W. A prospective observational study on the feasibility of subumbilical laparoscopic procedures under epidural anesthesia in sedated spontaneously breathing infants with a natural airway. Paediatr Anaesth. (2022) 32:49–55. doi: 10.1111/pan.14302, PMID: 34582607 PMC9292952

[11] Zaric D Axelsson K Philipson L Nydahl PA Larsson P Jansson JR. Blockade of the abdominal muscles measured by EMG during lumbar epidural analgesia with ropivacaine--a double-blind study. Acta Anaesthesiol Scand. (1993) 37:274–80. doi: 10.1111/j.1399-6576.1993.tb03715.x, PMID: 8517105

[12] Wiegele M Marhofer P Lönnqvist PA. Caudal epidural blocks in paediatric patients: a review and practical considerations. Br J Anaesth. (2019) 122:509–17. doi: 10.1016/j.bja.2018.11.030, PMID: 30857607 PMC6435837

[13] Martini CH Boon M Bevers RF Aarts LP Dahan A. Evaluation of surgical conditions during laparoscopic surgery in patients with moderate vs deep neuromuscular block. Br J Anaesth. (2014) 112:498–505. doi: 10.1093/bja/aet377, PMID: 24240315

[14] Boon M Martini CH Aarts LP Bevers RF Dahan A. Effect of variations in depth of neuromuscular blockade on rating of surgical conditions by surgeon and anesthesiologist in patients undergoing laparoscopic renal or prostatic surgery (BLISS trial): study protocol for a randomized controlled trial. Trials. (2013) 14:63. doi: 10.1186/1745-6215-14-63, PMID: 23452344 PMC3652756

[15] Honing GHM Martini CH Olofsen E Bevers RFM Huurman VAL Alwayn IPJ. Deep neuromuscular block does not improve surgical conditions in patients receiving sevoflurane anaesthesia for laparoscopic renal surgery. Br J Anaesth. (2021) 126:377–85. doi: 10.1016/j.bja.2020.09.024, PMID: 33092803 PMC7572301

[16] Özdemir-van Brunschot DMD Braat AE van der Jagt MFP Scheffer GJ Martini CH Langenhuijsen JF. Deep neuromuscular blockade improves surgical conditions during low-pressure pneumoperitoneum laparoscopic donor nephrectomy. Surg Endosc. (2018) 32:245–51. doi: 10.1007/s00464-017-5670-2, PMID: 28643056 PMC5770501

[17] Bruintjes MH van Helden EV Braat AE Dahan A Scheffer GJ van Laarhoven CJ. Deep neuromuscular block to optimize surgical space conditions during laparoscopic surgery: a systematic review and meta-analysis. Br J Anaesth. (2017) 118:834–42. doi: 10.1093/bja/aex116 28575335

[18] Blobner M Frick CG Stäuble RB Feussner H Schaller SJ Unterbuchner C. Neuromuscular blockade improves surgical conditions (NISCO). Surg Endosc. (2015) 29:627–36. doi: 10.1007/s00464-014-3711-7, PMID: 25125097

[19] Rosenberg J Herring WJ Blobner M Mulier JP Rahe-Meyer N Woo T. Deep neuromuscular blockade improves laparoscopic surgical conditions: a randomized, controlled study. Adv Ther. (2017) 34:925–36. doi: 10.1007/s12325-017-0495-x 28251555

[20] Dubois PE Putz L Jamart J Marotta ML Gourdin M Donnez O. Deep neuromuscular block improves surgical conditions during laparoscopic hysterectomy: a randomised controlled trial. Eur J Anaesthesiol. (2014) 31:430–6. doi: 10.1097/EJA.0000000000000094, PMID: 24809482

[21] Madsen MV Gätke MR Springborg HH Rosenberg J Lund J Istre O. Optimising abdominal space with deep neuromuscular blockade in gynaecologic laparoscopy--a randomised, blinded crossover study. Acta Anaesthesiol Scand. (2015) 59:441–7. doi: 10.1111/aas.12493, PMID: 25789421

[22] Tulgar S Boga I Cakiroglu B Thomas DT. Short-lasting pediatric laparoscopic surgery: are muscle relaxants necessary? Endotracheal intubation vs. laryngeal mask airway. J Pediatr Surg. (2017) 52:1705–10. doi: 10.1016/j.jpedsurg.2017.02.010, PMID: 28249684

[23] Ahiskalioglu A İnce İ Ahiskalioglu EO Oral A Aksoy M Yiğiter M. Is neuromuscular blocker necessary in pediatric patients undergoing laparoscopic inguinal hernia repair with percutaneous internal ring suturing? Eur J Pediatr Surg. (2017) 27:263–8. doi: 10.1055/s-0036-1587329 27548910

[24] Sauer M Stahn A Soltesz S Noeldge-Schomburg G Mencke T. The influence of residual neuromuscular block on the incidence of critical respiratory events. A randomised, prospective, placebo-controlled trial. Eur J Anaesthesiol. (2011) 28:842–8. doi: 10.1097/EJA.0b013e328345cd11, PMID: 21455074

[25] Murphy GS Szokol JW Avram MJ Greenberg SB Shear T Vender JS. Postoperative residual neuromuscular blockade is associated with impaired clinical recovery. Anesth Analg. (2013) 117:133–41. doi: 10.1213/ANE.0b013e3182742e75, PMID: 23337416

[26] Butterly A Bittner EA George E Sandberg WS Eikermann M Schmidt U. Postoperative residual curarization from intermediate-acting neuromuscular blocking agents delays recovery room discharge. Br J Anaesth. (2010) 105:304–9. doi: 10.1093/bja/aeq157, PMID: 20576632

[27] Kirmeier E Eriksson LI Lewald H Jonsson Fagerlund M Hoeft A Hollmann M. Post-anaesthesia pulmonary complications after use of muscle relaxants (POPULAR): a multicentre, prospective observational study. Lancet Respir Med. (2019) 7:129–40. doi: 10.1016/S2213-2600(18)30294-7, PMID: 30224322

[28] Hammer M Santer P Schaefer MS Althoff FC Wongtangman K Frey UH. Supraglottic airway device versus tracheal intubation and the risk of emergent postoperative intubation after general anaesthesia in adults: a retrospective cohort study. Br J Anaesth. (2021) 126:738–45. doi: 10.1016/j.bja.2020.10.040 33341223 PMC8014944

[29] Parker DF Round JM Sacco P Jones DA. A cross-sectional survey of upper and lower limb strength in boys and girls during childhood and adolescence. Ann Hum Biol. (1990) 17:199–211. doi: 10.1080/03014469000000962, PMID: 2337325

[30] Veneziano G Iliev P Tripi J Martin D Aldrink J Bhalla T. Continuous chloroprocaine infusion for thoracic and caudal epidurals as a postoperative analgesia modality in neonates, infants, and children. Paediatr Anaesth. (2016) 26:84–91. doi: 10.1111/pan.12807, PMID: 26530835

